# News Items

**Published:** 1885-10

**Authors:** 


### NEWS ITEMS.

THE American Academy of Medicine will meet in New York City on October 28th and 29th.

THE New York State Medical Association will hold its annual session at the Murray Hill Hotel, New York City, on the 17th, 18th and 19th of October.

